# Acute Immobilization Stress Modulate GABA Release from Rat Olfactory Bulb: Involvement of Endocannabinoids—Cannabinoids and Acute Stress Modulate GABA Release

**DOI:** 10.1155/2011/529851

**Published:** 2011-07-12

**Authors:** Alejandra Delgado, Erica H. Jaffé

**Affiliations:** Laboratorio Neuroquimica, Apartado 20632, CBB, IVIC, Caracas 1020 A, Venezuela

## Abstract

We studied the effects of cannabinoids and acute immobilization stress on the regulation of GABA release in the olfactory bulb. Glutamate-stimulated 3H-GABA release was measured in superfused slices. We report that cannabinoids as WIN55, 212-2, methanandamide, and 2-arachidonoylglycerol were able to inhibit glutamate- and KCl-stimulated 3H-GABA release. This effect was blocked by the CB1 antagonist AM281. On the other hand, acute stress was able *per se* to increase endocannabinoid activity. This effect was evident since the inhibition of stimulated GABA release by acute stress was reversed with AM281 and tetrahydrolipstatin. Inhibition of the endocannabinoid transport or its catabolism showed reduction of GABA release, antagonized by AM281 in control and stressed animals. These results point to endocannabinoids as inhibitory modulators of GABA release in the olfactory bulb acting through an autocrine mechanism. Apparently, stress increases the endocannabinoid system, modulating GABAergic synaptic function in a primary sensory organ.

## 1. Introduction

Cannabinoids comprise a family of lipids/eicosanoids, derived from marijuana/Hashish (Cannabis sativa). They may also be endogenous to animals (endocannabinoids) or synthetically produced (cannabimimetic) [1, 2]. Cannabinoids play a critical neuromodulatory role in the central and peripheral nervous system, as well as in the immune system, being an emerging therapeutic target for several disorders as addiction, obesity, nauseas and vomiting, pain, mental disorders, spasticity, glaucoma, and others [1, 3–5]. Apparently, they may also be associated with an old and widely used drug as acetaminophen/paracetamol, which is metabolized to a bioactive cannabimimetic drug AM404, an inhibitor of endocanabinoid uptake [6, 7].

Endocannabinoids (eCB) participate in intracellular signaling, being synthesized upon stimulation and increase of citoplasmatic Ca^2+^, exerting their effect through at least two G protein coupled receptors, CB1 and CB2, and the vanilloid receptor (TRPV1) [5, 8–15]. It has been shown that different cannabinoids are able to inhibit, through activation of CB1 receptors, the release of neurotransmitters, as GABA, glutamate (Glu), acetylcholine, noradrenalin, and dopamine from several SNS structures as cerebellum, hippocampus, striatum, substancia nigra, cortex, and so forth [2, 16]. The fatty acid amide hydrolase (FAAH) and monoacylglycerol lipase (MGL) are important enzymes in the catabolism of endocannabinoids and have been located in several structures of the CNS and specifically FAAH in the olfactory bulb [5, 17, 18]. Also the transporter of eCB is important in regulating the activity of the cannabinoid system in the CNS [5, 19, 20]. 

On the other hand, stress is the primary cause for fear or anxiety state which is an adaptive response to a threat. It is still controversial whether cannabinoids regulate stress, emotion, and mood disorders only through central mechanism by regulating the hypothalamus-pituitary-adrenal (HPA) axis and other CNS regions [5, 21] or also in combination with more peripheral levels as sensory gating mechanisms of odor, taste, touch, and so forth [22–25]. Several reports have shown that cannabinoids are able to modulate the release of neurotransmitters (5HT, GABA, Glutamate, Opioids, etc.) at the level of CNS nuclei as cerebral cortex, hippocampus, N. Accumbens [2, 5, 16] which are associated to stress and anxiety response [13, 26, 27]. At the level of the hypothalamus-pituitary–adrenal axis, the primary level to stress response, an important association between endocannabinoid activity and reduction of the stress response of this axis has been observed [21]. At the periphery, CB1 receptors have been shown to be present at several levels and are able to modulate nociception [28–30]. 

Endogenous and exogenous cannabinoids are able to modulate sensory perception, mood, anxious states, stress, pain, and so forth [4, 5, 26, 31]. Depending of the dose and time frame of cannabinoids exposure, they may exert an anxiolytic or anxiogenic effect, but generally anxiolytic at low concentrations [4, 5, 26, 27]. However, no reports are presently available of the effect of stress and cannabinoids on the synaptic regulation in olfactory bulb and specifically the regulation of GABA release. GABA is an important neurotransmitter in this structure [32–34] being able to act as a sensory modulator. 

In the olfactory bulb, high density of CB1 and vanilloid receptor labeling have been described [8–10, 35] together with the presence of the enzyme, fatty acid amide hydrolase (FAAH) is one of the enzymes which catalyses the hydrolysis of endocannabinoids [17, 18], and one of its precursors N-arachidonoyl phosphatidylethanolamine [36].

 On the other hand it is known that GABAergic granule cells establish dendro-dendritic synapses with glutamatergic mitral cells at the level of the external plexiform layer (EPL) [37] and that 3H-GABA is released from granule cells using high K^+^ and glutamate stimulation in a Ca^2+^-dependent mode [38]. NMDA and non-NMDA receptors have been described in the olfactory bulb [33, 37] which mediated the Glu-stimulate release of 3H-GABA from granule cell dendrites from EPL of rat olfactory bulb [39].

 In the present work, we were interested to test whether immobilization stress and cannabinoids were able to regulate GABA neurotransmission in the olfactory bulb. Here we show for the first time that synthetic cannabinoids as methanandamide/met-arachidonylethanolamide (AEA), WIN55,212-2 (Win), and 2-arachidonylglicerol (2AG) are able to inhibit Glu- and K^+^-stimulated release of 3H-GABA in this preparation. It is important to point out that the use of the AEA in our experimental conditions was favored because of its potency and resistance to encocannabinoid metabolism [40, 41]. Inhibition of anandamide transport with AM404 [42, 43] and its catabolism through FAAH with URB597 [44–46] has a strong inhibitory effect on stimulated GABA release. Also an inhibitor of the synthesis of 2AG, tetrahydrolipstatin (THL) [47] increases significantly Glu-stimulated GABA release; these results point to an important endogenous effect of endocannabinoids on the GABAergic terminals in the olfactory bulb. Acute immobilization stress is able to induce the release of endocannabinoids inhibiting the Glu-stimulated GABA release, apparently through CB1 receptors.

## 2. Methods

### 2.1. Acute Stress

Male Sprague-Dawley rats from IVIC, weighing 250–300 g, were housed 4 per cage of the following size: 61 × 42 × 19 cm. The animal room had controlled temperature and a 12 hr light dark cycle (06:00–18:00). Animals were supplied with lab. Chow and water *ad libitum*. Control animals were kept in the animal quarters. The stressing procedures consisted in keeping the animals in a restraining cage for one hour in the morning. The restraining cage was a rectangular Plexiglas box (6 × 5 × 12 cm) with the tail-gate adjusted to keep the rat well contained without being able to turn from front to back.

All animal experiments were carried out in accordance with the NIH guide for the care and use of Laboratory animals. All efforts were made to minimize animal suffering and use the minimum necessary number of animals. 

#### 2.1.1. Slice Preparation and Microdissection

**Figure 1:**
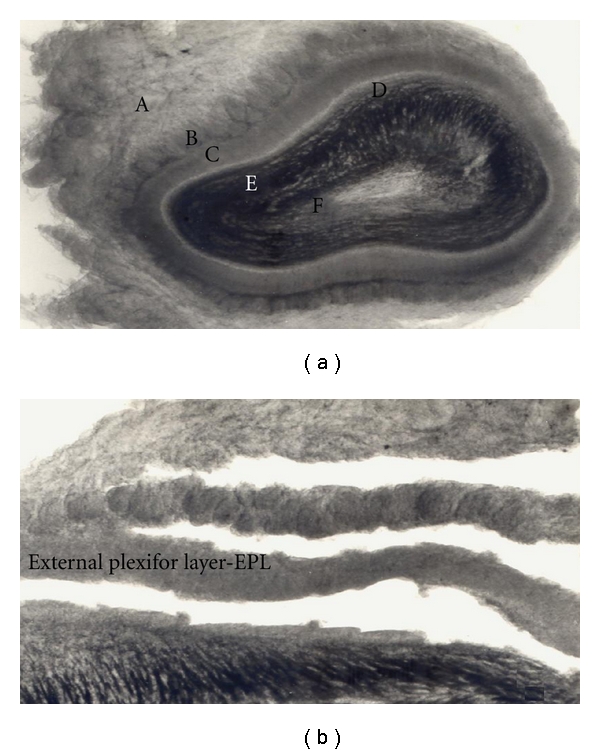
Microdissection of olfactory bulb slices. Rat Olfactory bulb slices were separated by free-hand microdissection using a dissection microscope equipped with a cold stage and used immediately for the release experiments. (a) Complete 400 *μ*m thick olfactory bulb slice, as seen under the dissection microscope. (b) Micro dissected layers of the bulb. A: Olfactory nerve layer. B: Glomerular layer. C: External plexiform layer (EPL). D: Mitral cell layer. E: Granule cell layer. F: Ependyma layer.

Male Sprague-Dawley rats, weighing 250–300 g, were quickly decapitated using a guillotine, and 0.4 mm slices of the olfactory bulb were obtained using a McIlwain tissue chopper. Slices were transferred to a Petri dish containing Krebs bicarbonate solution under continuous oxygenation with 95% 0_2_/5% CO_2_. The EPL, which contains high density of granule cell GABAergic dendrites [30], was separated by free-hand microdissection using a dissection microscope equipped with a cold stage and used immediately for the release experiments (Figure 1). All these steps were carried out at 4°C. 

#### 2.1.2. Release Studies

The method to study release was as described by Jaffé and Vaello [34]. Micro dissected EPL (Figure 1(b)) was preincubated in 1.5 mL Krebs bicarbonate solution at 25°C for 10 min. (in mM: KCl 2,5; KH_2_P0_4_ 1.25; MgCl 1, NaCl 125; CaCl_2_ 2; glucose 10, NaHC0_3_ 26; aminooxyacetic acid (AOAA) 10 *μ*M; and equilibrated with 0_2_/C0_2_ to pH 7.4). 3H-GABA was added to a final concentration of 0.1 *μ*M and incubation continued for 15 min under continuous oxygenation with 95% 0_2_/5% CO_2_. The tissue was then transferred to a 500 uL superfusion chambers, and superfused, at a rate of 500 *μ*L/min, with Krebs-bicarbonate solution under continuous oxygenation. During the first 35 min, the tissue sections were washed with the Krebs solution, and thereafter the superfusate samples were collected, at 2-minute intervals, using a fraction collector. Baseline level of 3H-GABA release was collected for 14 min, and 100 *μ*M glutamate or 15 mM KCl were added to the superfusate, for 4 min. to stimulate 3H-GABA release. Aliquot of 400 *μ*L was transferred to scintillation vials, and 2.6 mL of a scintillate solution, Aquasol (New England Nuclear), was added. Radioactivity was measured by liquid scintillation spectrometry. At the end of each experiment, the tissue was dissolved in triton X-100, and the radioactivity counted. 

The release of 3H-GABA was expressed as a percentage of the total amount of radioactivity remaining in the tissue, at the time of collection, released per minute. The evoked release is expressed as the ratio of the evoked release, during the highest 3 min. release during stimulation, divided by the basal release. Basal release was the mean value of the evoked release during 2 fractions before and after the complete release effect of stimulation, multiplied by 3. These values were treated with nonparametric statistical analysis using Mann-Whitney two-tailed rank sum test [48].

### 2.2. Measurement of Corticosterone

Levels of corticosterone in serum of control and stressed animals were measured using the commercial Kit for rat corticosterone EIA (dsl: diagnostic lab. Inc.USA).

### 2.3. Materials

KCl, KH_2_P0_4_, MgCl, NaCl, CaCl_2_, glucose, NaHC0_3_ were from Merk, aminooxyacetic acid (AOAA) was from Sigma-RBI. 4-Amino-n-[2,3-3H] butyric acid (3H- GABA) was purchased from GE-Amersham. Aquasol was from Perkin Elmers. All cannabinoid drugs and glutamate were from Sigma-RBI and prepared as stock solutions in DMSO as indicated by the provider.

## 3. Results

### 3.1. Levels of Corticosterone

Experimental animals were immobilized as described in methods. Control and experimental animals were sacrificed and blood taken for the measurement of corticosterone. The serum of stressed and control animals was stored at −20 until used with the corticosterone Kit. The levels of corticosterone in stressed rats were significantly increased when compared to control animals, being 161, 7 ± 17 ng/mL in control (12) and 333, 9 ± 37 ng/mL in stressed rats (12), with *P* < 0.001 for the Student *t* test statistical significance.

### 3.2. Modulation of the Release of 3H-GABA by Cannabinoids

**Figure 2:**
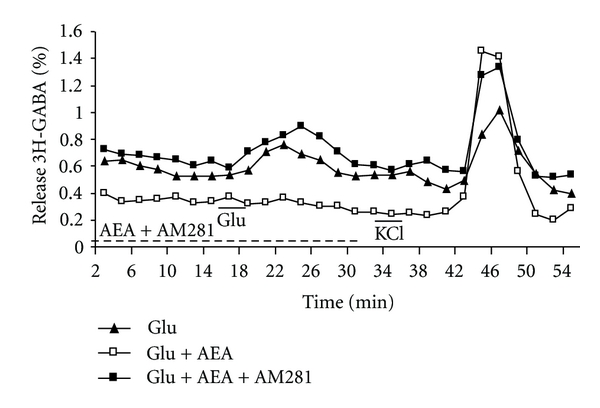
Glutamate stimulated 3H-GABA release from olfactory bulb: effect of cannabinoids. Micro dissected EPL was incubated with 0.1 *μ*M 3H-GABA and superfused with Krebs bicarbonate; after a wash of 35 min and 14 min of basal release, 100 *μ*M of Glu was added for 4 min followed after 22 min by 30 mM KCl stimulation (-▲-). The cannabinoid agonist methanandamide (AEA)(10 *μ*M) was added 14 min before, during, and 12 min after Glu stimulation (-□-). The CB1 antagonist AM281 (10 *μ*M) was added together with AEA in another set of experiments (-■-). Results are expressed as % of the total 3H-GABA remaining in the tissue at the time of collection, released per minute of one representative experiment.

**Figure 3:**
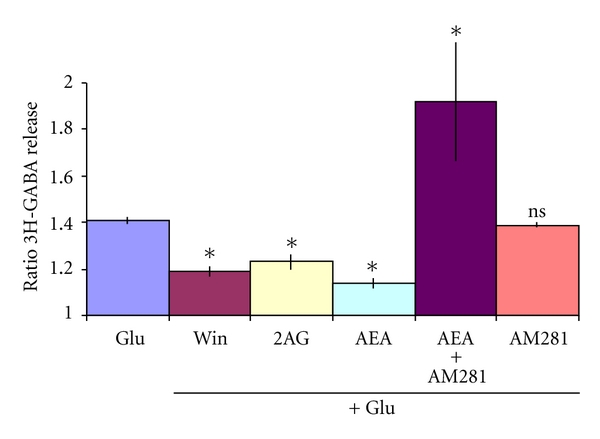
Effect of cannabinoid agonist and antagonist on Glu-stimulated 3H-GABA release. Micro dissected EPL was incubated with 0.1 *μ*M 3H-GABA and superfused with Krebs bicarbonate; after 49 min of superfusion 100 *μ*M of Glu was added for 4 min (Glu). The cannabinoid agonist WIN55, 212-2, 1 *μ*M (Win), 2-arachidonoylglycerol 10 *μ*M (2AG,) methanandamide, 10 *μ*M (AEA) were added 14 min before, during, and 12 min after Glu stimulation. The antagonist AM281 (1 *μ*M) was added alone or together with AEA. Results are expressed as a ratio of the % of 3H-GABA released as described in methods. *P < 0.01 compared to its Glu control, ns: not significant using Mann Whitney test.

Slices of the EPL layer of the olfactory bulb (Figure 1) were incubated with 0,1 *μ*M of 3H-GABA, and after a wash of 35 min, the basal release level was measured for 14 min and stimulated with 100 *μ*M glutamate (Glu) during 4 min. As shown in Figure 2, a small but significant increase of 3H-GABA release was obtained. A second control KCl stimulation (30 mM), as a test for tissue viability, was applied for 4 min, giving a robust release response. Perfusing the slices with 10 *μ*M of the cannabimimetic drug, AEA for 14 min before, during, and 12 min after the Glu stimulation reduced significantly the 3H-GABA release (Figures 2 and 3). The CB1 receptor antagonist AM281 [49, 50] when applied together with AEA was able to abolish the effect of AEA and even potentiate Glu-stimulated GABA release (Figures 2 and 3); however, AM281 alone did not show a significant effect on Glu-stimulated GABA release at a concentration of 1 uM. Even concentrations of the CB1 antagonist, as high as 10 uM, were not able to show an effect on stimulated GABA release (results not shown). Also Win (1uM) and 2AG (10 uM), two cannabinoid agonist, showed similar inhibition of the Glu-stimulated release.

**Figure 4:**
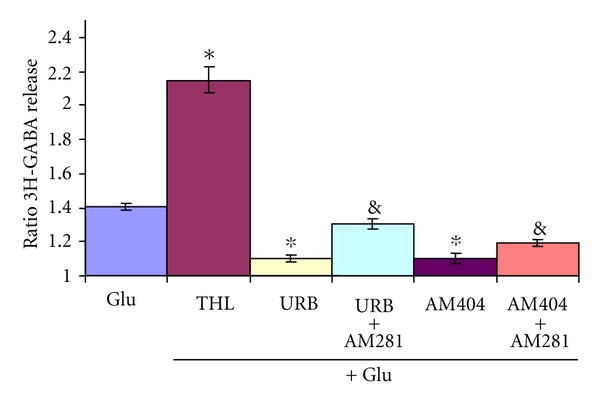
Effect of drugs acting on cannabinoid metabolism on Glu-stimulated 3H-GABA release. Micro dissected EPL was incubated with 0.1 *μ*M 3H-GABA and superfused with Krebs bicarbonate; after 49 min of superfusion, 100 *μ*M of Glu was added for 4 min (Glu). Tetrahydrolipostatin 0.5 uM (THL), an inhibitor of synthesis of 2AG, AM404 20 *μ*M, the inhibitor of anandamide transport, and URB597 250 nM, an inhibitor of the anandamide degrading enzyme FAAH, were added to the superfusing Krebs as in the agonist experiments with or without the antagonist AM281 (1 *μ*M). Results are expressed as a ratio of the % of 3H-GABA released as described in methods. *P < 0.01 compared to Glu control, &: significant compared to its control using Mann Whitney test.

Inhibition of GABA release was also observed when the transport of endocannabinoids was inhibited with 20 *μ*M AM404 [42, 44] (Figure 4) or with 250 *μ*M URB597, an inhibitor of the eCB degrading enzyme FAAH [44–46], (Figure 4). The inhibitory effects of AM404 and URB597 responses were reversed by the CB1 antagonist AM281 (1 *μ*M or 10 *μ*M) (Figure 4).

 Tetrahydrolipostatin (THL) 0,5 *μ*M, an inhibitor of the synthesis of 2AG, [47] showed a strong potentiating effect on Glu-stimulated GABA release (Figure 4).

**Figure 5:**
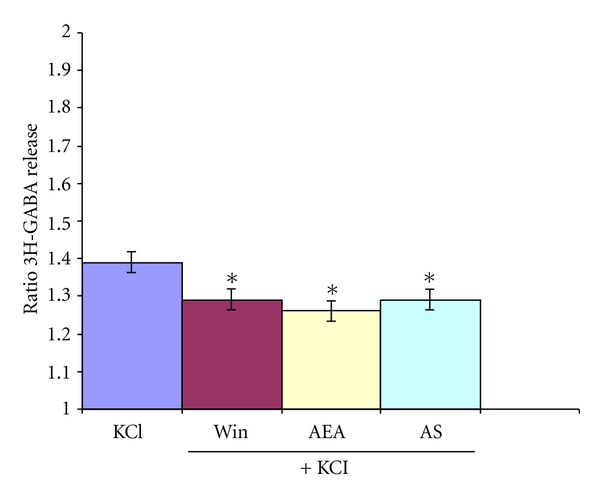
Release of KCl-stimulated 3H-GABA: effect of cannabinoid agonist in control and acute stressed rats. Micro dissected EPL was incubated with 0.1 *μ*M 3H-GABA and superfused with Krebs bicarbonate; after of 49 min of superfusion, 15 mM of KCl was added for 4 min. The cannabinoid agonist Win 1 *μ*M or methanandamide 10 *μ*M (AEA) was added 14 min before, during, and 12 min after KCl stimulation. Rats subjected to 1 hr. immobilization stress (Acute stress: AS) were sacrificed and olfactory bulb immediately used for experiments. Results are expressed as a ratio of the % of 3H-GABA released as described in methods. *P < 0.01 compared to control using Mann Whitney test.

When GABA release was unspecifically stimulated with 15 mM KCl, it was able to elicit a significant release of 3H-GABA which was inhibited by 2 cannabimimetic drugs as Win (1 *μ*M) or AEA (10 *μ*M) similar to the Glu-stimulated effect (Figure 5).

### 3.3. Modulation of the Release of 3H-GABA in Acute Stressed Animals

**Figure 6:**
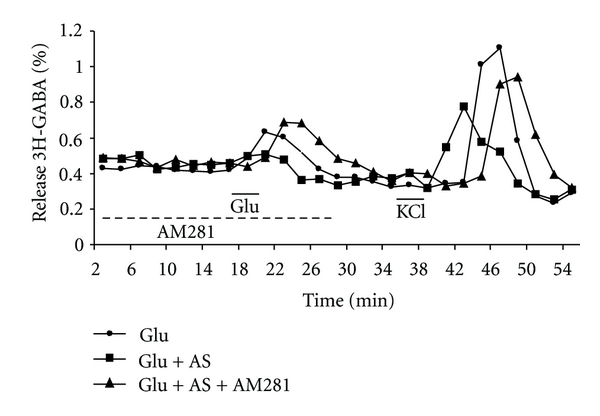
Glutamate-stimulated release of 3H-GABA: effect of cannabinoids in acute stressed rats. Rats subjected to 1 hr. immobilization stress (AS) were sacrificed and olfactory bulb immediately used for experiments. Micro dissected EPL of control and stressed animals were incubated with 0.1 *μ*M 3H-GABA and superfused with Krebs bicarbonate; after a wash of 35 min and 14 min of basal release, 100 *μ*M of Glu was added for 4 min followed after 22 min by a 30 mM KCl stimulation in control animals (-●-) and in acutely stressed one (-■-). The cannabinoid antagonist AM281 (5 *μ*M) was added 14 min before, during, and 12 min after Glu stimulation in stressed animals (-▲-). Results are expressed as % of the total 3H-GABA remaining in the tissue as described in methods of one representative experiment.

**Figure 7:**
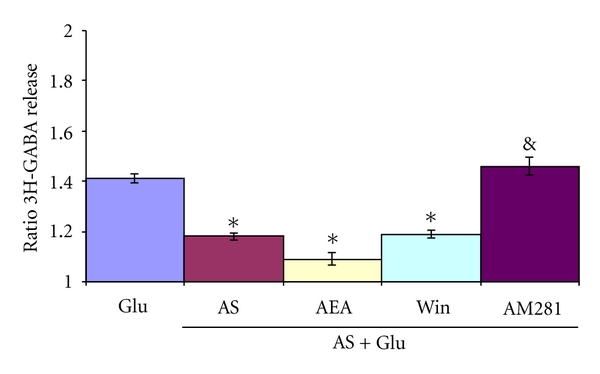
Glutamate-stimulated release of 3H-GABA from rat olfactory bulb: effect of acute stress and cannabinoids. Rats subjected to 1 hr. immobilization stress were sacrificed, and olfactory bulb immediately used for experiments. Micro dissected EPL of control and stressed animals were incubated with 0.1 *μ*M 3H-GABA and superfused with Krebs bicarbonate; after 49 min of superfusion, 100 *μ*M of Glu was added for 4 min in control (Glu) and acutely stressed (AS) animals. The cannabinoid agonist WIN55, 212-2, 1 *μ*M (Win), methanandamide, 10 *μ*M (AEA) was added 14 min before, during, and 12 min after Glu stimulation. The antagonist AM281 (5 *μ*M) was added during Glu stimulation. Results are expressed as a ratio of the % of 3H-GABA released as described in methods. *P < 0.01 compared to Glu control, and *P* < 0.01 compares to AS using Mann Whitney test.

Animals were immobilized for 1 hr. as described in methods. Immediately after the acute stress animals were sacrificed, slices were used for the release experiments. Control animals were not subjected to the stress procedure. As shown in Figures 6 and 7, acute stress, by its own, was able to inhibit significantly the Glu stimulated 3H-GABA release. This effect was reversed by 5*μ*M AM281 the CB1R antagonist (Figures 6 and 7).

**Figure 8:**
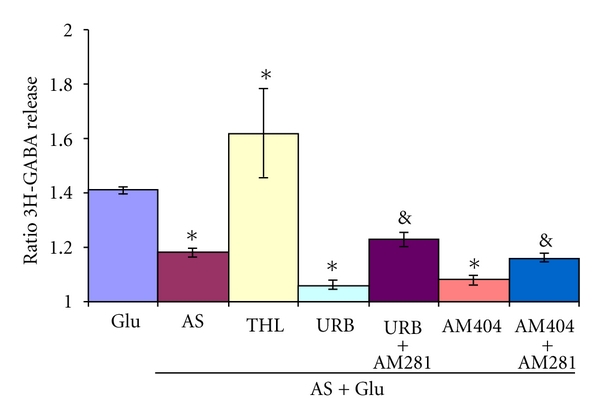
Effect of drugs acting on cannabinoid metabolism on Glu-stimulated 3H-GABA release of acutely stressed rats. Micro dissected EPL was incubated with 0.1 *μ*M 3H-GABA and superfused with Krebs bicarbonate; after 49 min of superfusion, 100 *μ*M of Glu was added for 4 min (Glu). Tetrahydrolipostatin 0,5 uM (THL), an inhibitor of synthesis of 2AG, AM404 20 *μ*M, the inhibitor of anandamide transport, and URB597 250 nM, an inhibitor of the anandamide degrading enzyme FAAH, were added to the superfusing Krebs as in the agonist experiments with or without the antagonist AM281 (5 *μ*M). Results are expressed as a ratio of the % of 3H-GABA released as described in methods. *P < 0.01 compared to Glu control, &: significant compared to its control using Mann Whitney test.

 When cannabinoid agonists AEA (10 *μ*M) and Win (1 *μ*M) were added 14 min before, during, and 12 min after the Glu-stimulation, a significant inhibition of Glu stimulated GABA release was observed but with no further increase of the inhibitory effect of stressed animals (Figure 7). However, inhibiting the transport or the catabolism of endocannabinoids with AM404 (20 *μ*M) or URB597, (250 *μ*M) respectively, did significantly enhance the inhibition of stress on 3H-GABA release (Figure 8). Both inhibitory responses were blocked when the CB1 antagonist AM281 (5 *μ*M) was applied before and during the Glu stimulation (Figure 8). 

Acute stress also diminished the KCl-(15 mM) stimulated release of 3H-GABA (Figure 5).

### 3.4. Modulation of Spontaneous Release of 3H-GABA

Spontaneous (basal) release of 3H-GABA was also modified in some conditions. So when endocannabinoids are increased with addition of AEA or when catabolism or transport is inhibited with URB597 or AM404, respectively, basal 3H-GABA release was significantly diminished (Table 1). Also the treatment with THL, the inhibitor of 2AG synthesis, increased significantly basal release of 3H-GABA release (Table 1).

## 4. Discussion

For centuries it has been known that cannabinoids have a wide range of effects in the CNS, modulating perception and mood. One of the earliest medical uses of cannabinoids has been in pain treatment and many studies have brought evidence for a central and peripheral action of cannabinoids on pain. This is consistent with the presence of CB1 and CB2 receptors at the level of CNS, spinal cord and peripheral sensory nerves, with a complex mechanism of action and interaction with other neuronal and immunological mediators [4, 5]. At the level of other sensory systems, as smell, little is known, though high to moderate levels of CB1 and TRPV1 cannabinoid receptors have been described in the olfactory bulb and olfactory epithelium [9–11, 35, 51]. The presence of the catabolizing enzyme FAHH has also been described in the olfactory bulb [17, 18]. Another evidence for the possible importance of cannabinoid in the OB and mood is the use, as a model of depression, of olfactory bulbectomy (OBX) which can be reverted with cannabimimetics substances [52, 53]. 

 In agreement with results obtained in other structures of the CNS [2, 5, 16], we demonstrate that cannabinoids and eCBs inhibit GABA release from granule cells of the olfactory bulb. In fact, here we show that synthetic cannabinoids, AEA and Win, or 2AG and eCB, are able to inhibit Glu- and KCl-stimulated release of 3H-GABA in this structure. These effects are blocked by the CB1 antagonist AM281, arguing in favor of the regulation of GABA release through CB1 receptors. Inhibition of the endocannabinoid transport with AM404, or of the metabolic enzyme FAAH with URB597, diminished Glu-stimulated GABA release, pointing to the presence of an endocanabinoid regulation in the olfactory bulb. Possibly, this inhibition is also mediated through CB1 receptors since the effect of AM404 and URB597 is partially reversed by the CB1 antagonist AM281. Other mechanisms beside the action of CB1 receptors cannot be excluded since eCBs may act at the level of other receptors or even as intracellular messengers [5, 54]. 

2AG is apparently also an important endogenous cannabin in the OB since the inhibition of the synthesis of 2AG with THL caused a strong increase in stimulated GABA release. 

We also show that spontaneous GABA release is inhibited by treatments that increase cannabinoids levels and is increased when the synthesis of 2AG is inhibited with THL. Thus beside the stimulated, “on demand”, release, there is also an important constitutive activity of eCB system, in the olfactory bulb under our experimental conditions. Such a dual mechanisms is however still controversial in other experimental systems [5].

**Figure 9:**
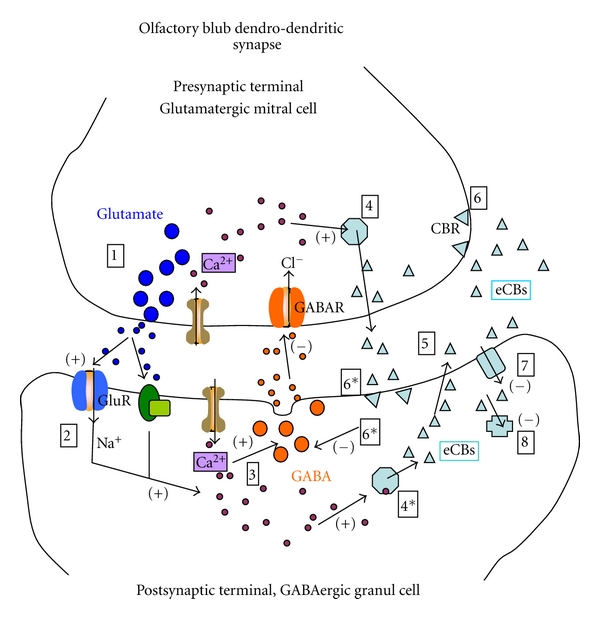
Schematic diagram of a reciprocal synapse from the EPL of the olfactory bulb. Stimulation of mitral cells release glutamate, depolarizing granule cells (1) through VGCC and/or NMDA/AMPA receptors (2) increasing cytoplasmic Ca^2+^. (3). This depolarization of granule cells induces exocytosis of GABA. On the other hand, the increase of intracellular Ca^2+^ or/and the activation of mGlu receptors (2-3) triggers the synthesis of endocannabinoids (anandamide/2AG) (4) which diffuse to the extracellular space (5) activating cannabinoid CB receptors (6) at the level of the granule cells and possible also the mitral cells terminals. This activation of CB receptors is able to inhibit GABA release (and eventually Glu release?). The eCB transporter (7) and the catabolic enzymes eliminate eCBs (8). Acute stress is apparently able to induce synthesis of eCB (4) and inhibit GABA release; this effect can be potentiated when the eCBs transporter (7) or the catabolizing enzyme (8) is blocked or when the synthesis of 2AG is inhibited with THL. NMDA/AMPA: inotropic glutamate receptors, mGluR: Metabotropic glutamate receptor, VGCC: Voltage-gated Ca^2+^ channels, AEAT: Arachidonyl ethanol amine transporter, CBR: Cannabinoid receptor, FAAH: Fatty acid amide hydrolase, GABA: *γ*-amino butyric acid, eCB: endocannabinoid, 2AG: 2-arachidonylglicerol, THL: tetrahydrolipostatin. *Modulator sites of acute immobilization stress.

It is interesting to point out that the effect of eCBs that we have described occurs on the same GABA terminal stimulated by Glu or KCl. This endocanabinoid action on the EPL can be compared to a presynaptic autoreceptor mechanism, described for many classical neurotransmitters [55] (Figure 9). In the olfactory bulb, GABA release, stimulated by the activation of inotropic (NMDA/AMPA) [37, 39], and metabotropic (mGlu1) [56] Glutamate receptors, could induce synthesis of endocannabinoids from the same GABA terminal (Figure 9). Upon synthesis and release, eCBs would act directly on their receptors, on the same terminal, inhibiting GABA release. This auto- modulation of endocannabinoids on the same terminal where it is synthesized and released would be comparable to a direct negative feedback as in an autocrine system [55] (Figure 9), which could inhibit GABA release together with the classical retrograde synaptic regulation described for cannabinoids [5, 57, 58]. 

At the level of the olfactory bulb, the precise localization of the CB receptors is not known and the possibility of their presence at the level of GABA as well as Glu terminals can not be excluded. Further studies are needed to evaluate the presence of an autocrine or/and a retrograde mechanism of endocannabinoid action at the level of the olfactory bulb. 

During acute stress, corticosterone levels were significantly increased when compared with the control animals, indicating activation of the hypothalamus-pituitary-adrenal (HPA) axis, since the activation of this system plays a pivotal role in the stress response [59]. It is not clear whether during stress, cannabinoids exert their effect only through central mechanisms or in combination with primary sensory and peripheral systems as odor, taste, and so forth, with a possible sensory gating mechanism [26, 60, 61]. The change in perception during different mood conditions and pathologies is an interesting regulatory mechanism to cope with stress situations.

The importance of eCB during stress or depression has been documented at several levels of the CNS [1, 4, 5]. At the level of the olfactory bulb, it has been shown that it may function as a gating mechanism during stress [61, 62], and indirect evidence of the importance of this sensory structure in mood is shown with the olfactory bulbectomy model of depression, which can be reverted with cannabimimetics [52, 53]. In the present work, we show that in the olfactory bulb, of acutely stressed rats, cannabinoids are probably able to modulate primary sensory input through inhibition of GABA release from granule cell dendrites (Figure 9). Thus, at the level of the olfactory bulb, acute stress, by its own, was able to inhibit GABA release, an effect reversed by the CB1 antagonist AM281, pointing to eCB activation in the olfactory bulb under our experimental conditions. No additional significant changes in GABA release were caused by cannabinoid agonists. However, when the catabolism or the transport of endocannabinoids was blocked with the CB1 antagonist, a potentiation of the stress effect on GABA release was observed suggesting a modulation at the level of increased synthesis or decreased uptake and metabolism of eCB during acute stress.

The endogenous inhibitory activity of cannabinoids during acute stress, at the level of the olfactory bulb, can also be seen with the inhibition of the synthesis of 2 AG with THL, which strongly increased the stimulated release of GABA. Apparently, during acute stress the increased eCB levels are able to inhibit GABA release through CB1 receptors and in this way act as a gating mechanism and change odor perception.

In control experiments, Glu-stimulated GABA release, was not affected by AM281, even at a high concentration, 10 uM. However, in the presence of AEA, which inhibited Glu-stimulated GABA release, AM281 at a lower concentration of 1 *μ*M reverted the effect of AEA, restoring and even increasing GABA release above control levels. This potentiation of GABA release could be explained in terms of some unknown interaction between AEA and AM281 perhaps involving an inverse agonist action of AM281 [5, 63].

The endocannabinoid metabolism is apparently constitutive in regulating GABA release since treatments that increase eCB levels as URB597 and AM404 inhibited spontaneous GABA release. Again as in the experiments with stimulated GABA release, AM281 showed an inverse effect on spontaneous release; at high concentrations as 10 uM it inhibited spontaneous GABA release, behaving as an agonist and not an antagonist probably by acting at other modulatory sites [2, 5, 12, 13]. Nevertheless, a very clear effect of a spontaneous synthesis of the eCB 2AG was demonstrated through the potentiation of the spontaneous release of GABA in the presence of THL, the inhibitor of the diacylglycerol lipase, a pivotal enzyme in the synthesis of 2AG [47]. These findings would indicate that in the olfactory bulb a tonic release of endocannabinoids could be observed in control and stressed animals, similar to what has been described in other preparations, as in CB1 receptor transfected cultured hippocampal neurons [5, 64]. 

Apparently, in olfactory bulb, endocannabinoids release, activated during acute stress, inhibits GABA release, thus modulating the primary sensory input of odor to higher levels of the CNS. In conclusion, endocannabinoids and stress are able to modulate GABAergic synaptic function at a sensory level (olfactory bulb). This modulation would be through an autocrine regulation acting through CB1 receptors.

## Figures and Tables

**Table 1:** Effect of cannabinoids and acute stress on basal 3H-GABA release. Micro dissected EPL was incubated with 0.1 *μ*M 3H-GABA and superfused with Krebs bicarbonate; after 47 min of superfusion a basal release was reached. Basal release is a % of release and was measured as described in methods and is the median with 95% confidence interval of experiments. The abbreviations of the drugs are as described above.

Control	0.50 (0.38–0.65) *n* : 65
AEA, 10 uM	0.41* (0.31–0.53) *n* : 34
Win, 1 uM	0.55^ns^ (0.38–0.59) *n* : 22
AM281, 10 uM	0.35* (0.3–0.47) *n* : 10
URB597, 250 uM	0.34* (0.31–0.39) *n* : 15
AM 404, 20 uM	0.37* (0.27–0.42) *n* : 16
THL, 0.5 uM	0.94* (0.61–1.06) *n* : 6
2AG, 10 uM	0.53^ns^ (0.41–0.6) *n* : 26
Acute Stress	0.42^ns^ (0.36–0.54) *n* : 30
